# Rapidly and exactly determining postharvest dry soybean seed quality based on machine vision technology

**DOI:** 10.1038/s41598-019-53796-w

**Published:** 2019-11-20

**Authors:** Ping Lin, Li Xiaoli, Du Li, Shanchao Jiang, Zhiyong Zou, Qun Lu, Yongming Chen

**Affiliations:** 1 College of Electrical Engineering, Yancheng Institute of Technology, Yancheng, Jiangsu China; 2 College of Biosystems Engineering and Food Science, Zhejiang University, Hangzhou, Zhejiang China; 3 College of Mechanical and Electrical Engineering, Sichuan Agricultural University, Ya’an, Sichuan China

**Keywords:** Plant sciences, Electrical and electronic engineering

## Abstract

The development of machine vision-based technologies to replace human labor for rapid and exact detection of agricultural product quality has received extensive attention. In this study, we describe a low-rank representation of jointly multi-modal bag-of-feature (JMBoF) classification framework for inspecting the appearance quality of postharvest dry soybean seeds. Two categories of speeded-up robust features and spatial layout of L*a*b* color features are extracted to characterize the dry soybean seed kernel. The bag-of-feature model is used to generate a visual dictionary descriptor from the above two features, respectively. In order to exactly represent the image characteristics, we introduce the low-rank representation (LRR) method to eliminate the redundant information from the long joint two kinds of modal dictionary descriptors. The multiclass support vector machine algorithm is used to classify the encoding LRR of the jointly multi-modal bag of features. We validate our JMBoF classification algorithm on the soybean seed image dataset. The proposed method significantly outperforms the state-of-the-art single-modal bag of features methods in the literature, which could contribute in the future as a significant and valuable technology in postharvest dry soybean seed classification procedure.

## Introduction

Soybean (Glycine max (L.) Merrill) is known as the “golden bean”. It is rich in amino acids, vitamins, minerals and fat, and is the most important source of protein concentrate and vegetable oil. The soybean seed quality can be measured in several ways, including the Raman spectroscopy^1,2^, near-infrared spectroscopy^3,4^, terahertz spectroscopy^5,6^, high-performance liquid chromatography-mass spectrometry^7,8^, capillary electrophoresis mass spectrometry^9,10^, scanning electron microscope^11,12^ and nuclear magnetic resonance^13,14^ techniques. These techniques can be used to discriminate the quality by measuring the chemical and nutritional ingredients of soybean seeds. The weaknesses of these methods are that it is time-consuming and needs complex experimental pretreatment, as well as they are not suitable for detecting the appearance parameter. The soybean seed product quality factor, price, and marketability are directly affected by the appearance quality. Besides, the defective soybean seeds often cause disease and can not grow into healthy plants. The first important step in the fine process and storage of soybean and its products is to screen out the seed samples with a poor appearance. Human and machine vision techniques can be employed to grade the seed according to the appearance of soybean seeds. The human vision-based grading method is time-consuming, low speed and efficiency for sorting a large amount of seeds. The machine vision-based technique has received much attention for food appearance quality assessment due to the non-destructive, low cost and high accuracy and efficiency compared with other detection and analysis techniques^15^.

In recent years, several artificial intelligent systems based on machine vision and machine learning technologies are presented for detection and discrimination of soybean seed quality. For example, Ahmad *et al*. conducted the experiment based on a knowledge domain using the color information for the classification of asymptomatic and symptomatic soybean seeds^16^. Shatadal & Tan trained a feed-forward neural network using RGB color features to classify the soybean seeds into sound, heat–damaged, green–frost–damaged and stink–bug–damaged categories^17^. Liu *et al*. extracted the L*a*b* color features, three texture features of energy, entropy and contrast and eight shape features of perimeter, area, circularity, elongation, compactness, eccentricity, elliptic axle ratio and equivalent diameter as the input of BP artificial neural network and set up a three layers classifier for sorting six categories -mildewed, insect-damage, broken, skin-damaged, partly detective and normal soybean kernels^18^. These previous methods used global visual characteristics of color, morphology, and texture to describe the soybean seeds. The global features usually contain an amount of invalid background information, and the local detailed information is easy to be masked by using them. The introduction of invalid features and the loss of effective detailed discrimination information will inevitably affect the performance of the classification model, thus affecting the final recognition accuracy. The defective soybean seed features often appear in the local image, even in the small local ranges. Compared with using the global features to describe the defective soybean seeds, effective local image features can be used as a key means to distinguish the quality of soybean seeds. Therefore, it is necessary to develop a new local feature algorithm to further improve the classification accuracy of soybean seeds.

In recent years, the state-of-the-art technologies of low-level local visual feature representation based on the bag-of-feature model showed great potential in object recognition. The BOF method who is derived from the document analysis method converts the low-level local image features to visual word features to represent the image property. Murat Olgun *et al*. (2016) used the BoF of dense scale invariant features to represent the wheat grain varieties^19^. Xiao *et al*. (2018) introduced a support vector machine (SVM) classifier for classifying four kinds of important southern vegetable pests based on scale-invariant feature transform (SIFT) BoF visual vocabulary^20^. The above investigations only used one kind of BoF visual dictionary, which is hard to fully express the complex agricultural objects. Abozar Nasirahmadi *et al*. presented a bag of feature model joining Harris, Harrise-Laplace, Hessian, Hessian-Laplace and maximally stable extremal regions key point detectors along with a scale invariant feature transform descriptor for classification of sweet and bitter almond varieties^21^. Although several local feature algorithms have been implemented for improving the performance of the agricultural product quality detection system, there is little study aimed at the detection quality of soybean seed.

In this paper, we intend to use the BoF-based algorithms to validate the effect of soybean seed image classification. Besides, the simple combination of multiple features will inevitably lead to redundancy of features to represent an image, and to a certain extent affect the performance of the classifier in the final process of feature recognition. To further improve the performance of intelligent recognition systems, this paper proposes a low-rank representation (LRR) algorithm^22,23^ to find the lowest rank representation among the long and distinct kinds of features in subspace. The method can organically merge the distinct category of semantic dictionaries by a generation of the new low-dimensional descriptors in low-rank subspace and eliminate the influence of irrelevant semantic dictionary information in such space.

The objective of this study was to exploit a low-rank representation of jointly multi-modal BoF (JMBoF) classification framework for exactly, non-destructively and fast identifying the quality of soybean seeds. The rest of the paper is organized as follows: firstly, the experimental materials and devices used to capture the images are introduced; secondly, the JMBoF-related methods for inspecting the dry soybean seed quality are presented; thirdly, the experimental results are shown and discussed; Finally, the conclusions are drawn.

## Experiments

### Experimental materials

**Figure 1:**
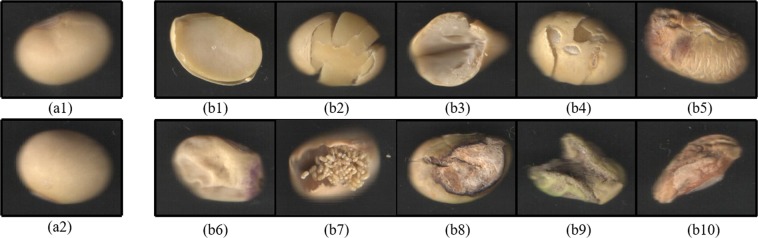
Images of different appearance quality of soybean seeds are applied to the classification investigation. Two good soybean seed sample images (**a1,a2**) are shown on the left side. Other two kinds of (**b1**) cotyledon-lacked, (**b2**) physically damaged, (**b3**) naturally cracked, (**b4**) testa-damaged, (**b5**) hull-shriveled, (**b6**) cotyledon-atrophic, (**b7**) worm-bitten, (**b8**) testa-decayed, (**b9**) cotyledon-mouldy and (**b10**) heteromorphic soybean seed images are shown on the right side.

The soybean seeds for the experiment were purchased from the local market. There are ten kinds of cotyledon-lacked, physically damaged, naturally cracked, testa-damaged, coat shriveled, cotyledon-atrophic, worm-bitten, testa-decayed, cotyledon-moldy and heteromorphic defective soybean seed samples (see Fig. 1), where the physically damaged means that the seed coat and cotyledons are split after the seed kernel is physically or mechanically squeezed. The cotyledon-lacked, physically damaged, naturally cracked and testa-damaged seed kernels without the protection of the outermost hull are prone to mildew after long-term storage. The severely hull-shriveled and cotyledon-atrophic seed kernels are considered to be malnourished, thus affecting human health and related product quality. Ingestion of the worm-bitten, testa-decayed, cotyledon-moldy and heteromorphic seeds can impair the health of humans and animals. Therefore, it is necessary and important to grade soybean seeds in terms of their appearance features. In this experiment, we attempt to automatically discriminate three grades of good, moderate and unhealthy soybean seeds in terms of their appearance quality. A good soybean seed comprises approximately 8% seed coat (or hull, or testa), 90% cotyledons and 2% Embryonic axis (including plumula, hypocotyl, and radicle)^24^. The good appearance features indicate that the seed coat is intact and smooth, as well as the cotyledons are plump, which will be good for the health of humans and animals (see Fig. 1(a1,a2)). The moderate one indicates that the seed coat is broken, the cotyledon is cracked, or the cotyledon is slightly shriveled, but it does not harm the health of humans and animals (see Fig. 1(b1–4)); The unhealthy one indicates that the seed coat or cotyledon is severely shriveled, cotyledon-atrophic, worm-bitten, testa-decayed, cotyledon-moldy or heteromorphic, which will damage the health of humans and animals after consumption (see Fig. 1(b5–10)). There are 843 soybean seeds used for the test. Each type has 281 samples. The training set contains 70% randomly selected samples, and the remaining 30% is used for test purposes.

### Experimental devices

The visual spectral imaging device (Perfection V850 Pro, Epson, US) is used to capture the image of the soybean seeds. The principal parts of the imaging system comprise black absorption cover, transparent flat glass plate, motor-driven shifting electronic platform, charge-coupled imaging device (CCD), black box, communication cable, and computer machine. Each soybean sample is laid at equal intervals on the transparent glass panel. Then the black absorption cover is placed horizontally above the samples as the image background. The motor-driven shifting platform carries the shifting linear light and shifting mirror. The shifting linear source emits the linear beams to the sample surface through the transparent flat glass plate. The sample reflects the light beams to the shifting mirror, and then the beams from the shifting mirror are reflected to the fixed mirror. Finally, the CCD collects the linear sample spectra transmitted from the fixed mirror. Compared with the traditional camera shooting technology, the motor-driven shifting electronic shooting platform to capture the photograph can ensure that each soybean seed in any position of the photograph is uniform. The imaging devices are fixed in a closed black box which can block the effects of external lighting. A communication cable is used to connect with the outside computer machine with the inside imaging devices. Each original captured photograph contains 20 kernels, in which each kernel image is automatically separated and stored to the disk.

## Methods

### Color space conversion

L*a*b* is a color space specified by the International Commission on Illumination (CIE)^25^, where L* is for the lightness and a* and b* are for the green–red and blue-yellow color components, respectively. The L*a*b* color space not only contains all the gamut of RGB color space but also expresses a part of color space that the RGB can not do. The RGB color space can not be directly transferred to L*a*b* color space. It takes two steps to implement the conversion. The RGB color space firstly must be transformed into a specific CIE XYZ color space^26^,

1 $$[\begin{array}{c}X\\ Y\\ Z\end{array}]=[\begin{array}{ccc}0.4124 & 0.3576 & 0.1805\\ 0.2126 & 0.7152 & 0.0722\\ 0.0193 & 0.1192 & 0.9505\end{array}]\,[\begin{array}{c}R\\ G\\ B\end{array}]$$

The L*a*b* color space is further defined relative to the tristimulus values of the reference white point (*X*_*n*_, *Y*_*n*_, *Z*_*n*_) of the XYZ space from which they were converted:

2 $$[\begin{array}{c}{L}^{\ast }\\ {a}^{\ast }\\ {b}^{\ast }\end{array}]=[\begin{array}{c}116f(Y/{Y}_{n})-16\\ 500(f(X/{X}_{n})-f(Y/{Y}_{n}))\\ 200(f(Y/{Y}_{n})-f(Z/{Z}_{n}))\end{array}]$$

where,

3 $$f(t)=\{\begin{array}{ll}\sqrt[3]{t} & {\rm{if}}\,t > {\rm{0.0089}}\\ {\rm{7.7870}}t+{\rm{0.1379}} & {\rm{otherwise}}\end{array}$$

**Figure 2:**
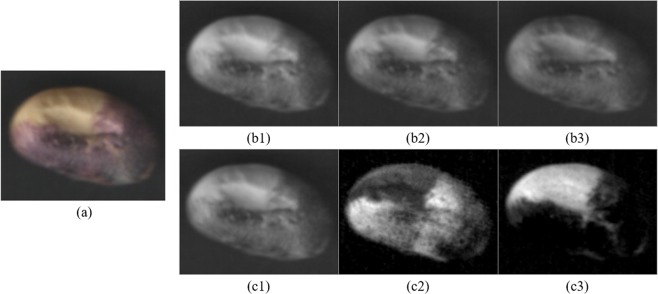
Color space conversion from RGB to CIE L*a*b* for easily quantifying the visual differences between colors. (**a**) A RGB image of soybean seed is decomposed into (**b1**) red, (**b2**) green and (**b3**) blue color components as well as converted to (**c1**) L*, (**c2**) a* and (**c3**) b* color channels.

An instance of color space conversion from RGB to L*a*b* is shown in Fig. 2.

### SURF feature space descriptors

SURF is a successful algorithm for feature detection introduced by Bay^27^. The goal is to define unique and robust space descriptors of an image. The algorithm consists of the following three main steps:

1. Detect interest points (see Fig. 3(a)). It takes advantage of an integer approximation of the determinant of the Hessian blob detector, which can be calculated by three predefined integral operators. Its feature descriptor is based on the sum of the Haar wavelet response around the point of interest; The category of feature area can be determined by the sign (denoted by −1 and +1) of the Laplacian (i.e. the trace of the Hessian matrix)^28^.

**Figure 3:**
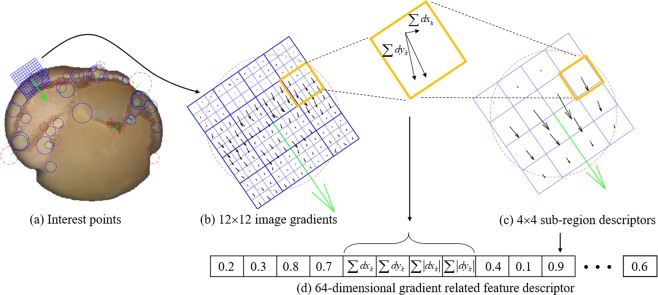
An example shows the detected (**a**) interest point: locations shown by the centres of circles, scales shown by the green radius of circles, dominant orientation shown by the direction of radius and the categories defined by the colors of circles. i.e., dark on yellow background (red dotted circles) or yellow beside dark region (blue solid circles); (**b**) image gradients computed by Haar wavelet responses at 12 × 12 regularly squared spaced sample points; (**c**) 4 × 4 subregion descriptors by calculated vector sum in 3 × 3 squared region of the Haar wavelet responses in horizontal and vertical directions, which are related to the dominant orientation of corresponding interest point shown by the green arrows and (**d**) a concatenated 64-dimensional gradient-related feature descriptor from 4 × 4 subregion underlying intensity structure.
2. Obtain gradient information in the subregion. The interest region is split into smaller 4 × 4 squared subregions aligned to the selected orientation (see Fig. 3(c)), and for each one, the Haar wavelet responses are extracted at 3 × 3 regularly spaced sample points (see Fig. 3(b)). The responses are multiplied by Gaussian gains to resist the deformations, noise, and translation.
3. Generate feature space descriptors. Concatenate 64-dimensional gradient related feature descriptor from each 4 × 4 local neighborhood subregion underlying intensity structure $(\sum d{x}_{k},\sum d{y}_{k},\sum |d{x}_{k}|,\sum |d{y}_{k}|)$ from each detected interest point to form the feature space descriptors (see Fig. 3(d)).

### Bag of feature model

BoF is a technique adapted to image categorization from the area of document categorization. Rather than using actual words as in document categorization. BoF algorithm uses image features like the visual words which are finally combined as the visual dictionary to represent an image^21,29^. To achieve this, it includes the following two main steps:

1. Extract a ‘bag’ of independent features. In this study, we extract two bags of features of the L*a*b* color features and the SURF features, where the L*a*b* color feature composes of two portions of the average of color components within 16 × 16 subregions of image and the corresponding spatial coordinates in an image where it was extracted.
2. Generate visual dictionary. The k-means clustering method is performed to cluster the feature vectors obtained in Step 1. The cluster center is defined as the visual word. All the visual words are collected to generate the visual dictionary. The number of clustering centers is the visual dictionary size. Thus, the low-level image features are quantized as the high-level semantic information to express the image content through the distribution of visual words.

### Jointly low-rank feature representation

In the aforementioned BoF method, the visual dictionary size will affect the features constituted by visual words on the interpretability of image content: the small size dictionary may not fully describe the image features, and too large size dictionary may cause redundant semantic expression. Besides, the new joint-modal features of SURF and L*a*b* will multiply the dimensionality of the dictionary, which will further increase the redundant semantic representation of features. To solve the issue, we firstly extract a large size of visual dictionary from images by setting a large number of dictionaries (numbers = 800) and then used the LRR method to eliminate the redundant semantic information to effectively express the image content. LRR supposes the high-dimensional data *Y* has low intrinsic dimensionality. In order to alleviate the curse of dimensionality, the original *Y* can be into two components of low-rank matrix *X* and sparse error matrix *E*:

4 $$\{\begin{array}{l}\mathop{\min }\limits_{X,E}({\Vert X\Vert }_{\ast }+\lambda {\Vert E\Vert }_{1})\\ \begin{array}{cc}s.t. & Y=X+E\end{array}\end{array}$$

where, $||\,\cdot \,|{|}_{\ast }$ denotes the nuclear norm, $||\,\cdot \,|{|}_{{\rm{1}}}$ denotes *L*1-norm and λ is a regularization parameter. The above optimization problem is essentially to find the optimal projection of high-dimensional data in a low-dimensional subspace. After removing the residual *E*, the compact visual dictionary set *X* will be used as the effective expression of the raw image^22,23^.

### Support vector machine regression

The algorithm of SVM^30,31^ is used to transform the input space into a high-dimensional Hibert space by nonlinear transformation, and then implement linear classification in this space. The SVM method assumes a set of training data Λ for a given set of points *N*:

5 $$\varLambda ={\{({x}_{i},{y}_{i})|{x}_{i}\in {R}^{p}\}}_{i=1}^{N}$$

where the predicted value *y*_*i*_ corresponds to the independent variable *x*_*i*_. The SVM method uses the kernel function *φ* to project the independent variable *x* into the high-dimensional feature space to establish the linear fitting function ${f}_{i}(x)={\omega }_{i}^{T}\phi (x)+{b}_{i}$. The equation can be set up by solving the following optimization problem:

6 $$\{\begin{array}{cc}\mathrm{minimize}: & \Omega (\omega ,{\zeta }_{j}^{i})=\frac{1}{2}{\Vert {\omega }_{i}\Vert }^{2}+\lambda \sum {\zeta }_{j}^{i}\\ \mathrm{subject\; to}: & 1-{\zeta }_{j}^{i}\le \hat{y}({\omega }_{i}^{T}\phi (x)+{b}_{i}),0\le {\zeta }_{j}^{i}\end{array}$$

where, *ω* is the normal vector to the hyperplane related to the complexity of the model; *λ* are adjustable parameters and ${\zeta }_{j}^{i}$ are relaxation variables.

### Statistical analysis

The *k*-fold cross-validation technique^31^ is used to assess the robustness of the established model for rapidly and exactly determining postharvest dry soybean seed quality. The training soybean samples are partitioned into *k* equal sized subsamples, where *k* is equal to the number of the training soybean samples. One of the *k* soybean samples is selected as the unknown validation data and the rest of the *k* − 1 soybean subsamples are used for training aim. Each of the partitioned *k* − 1 soybean subsample data will be trained exactly once to predict the rest of one data that was not used in estimation. The validation results are averaged over the *k* times to evaluate the classification performance of the fitting model.

### Algorithm overview

**Figure 4:**
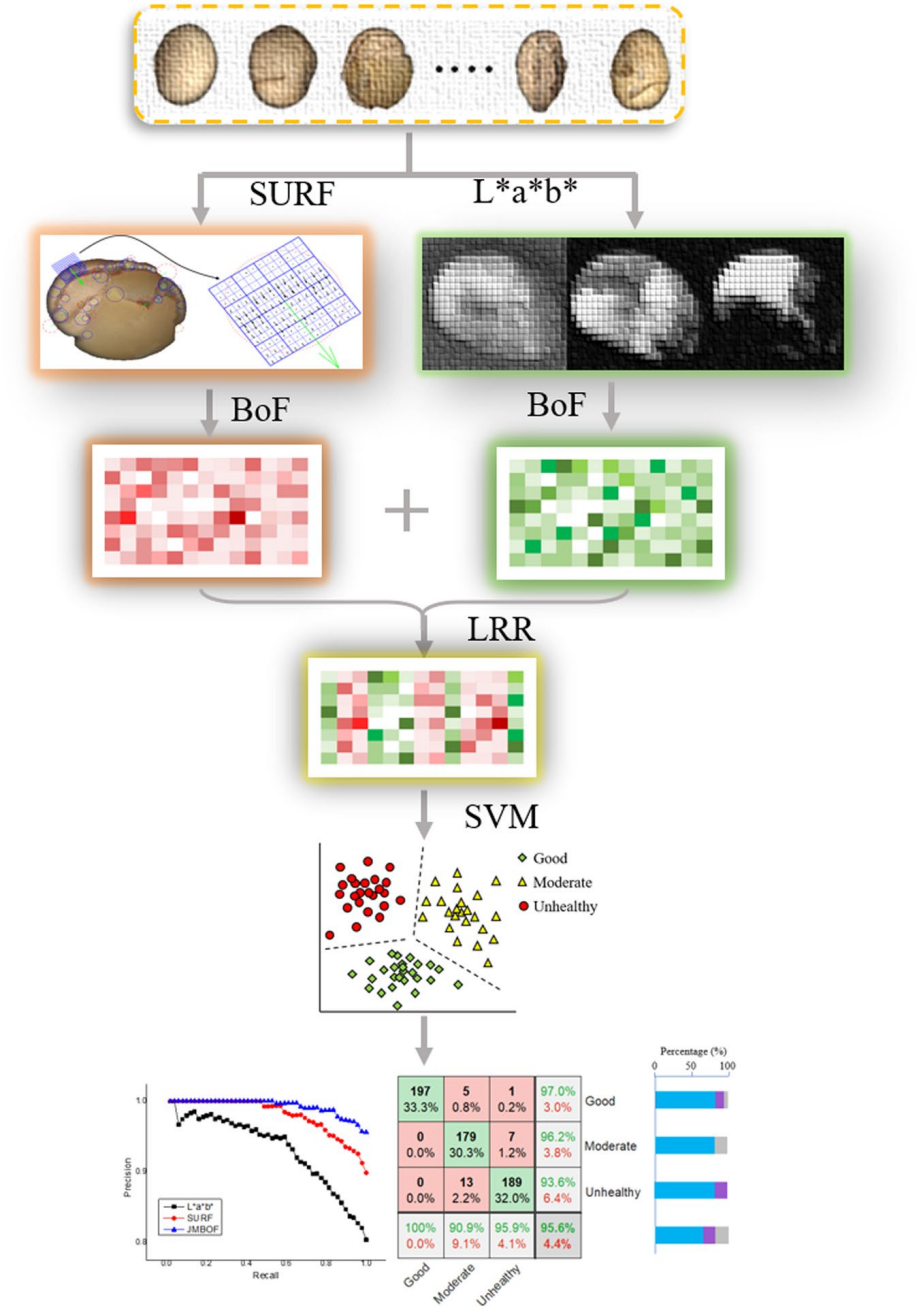
Schematic of low-rank representation of jointly multi-modal bag-of-feature classification framework for determining the quality of soybean seeds.

The schematic of the JMBoF classification framework for determining the quality of soybean seeds is shown in Fig. 4. The steps to implement our algorithm for the classification of soybean category are as follows

1. Extract the L*a*b* color components within 16 × 16 subregions of image and the corresponding spatial coordinates in an image where it was extracted.
2. Extract the SURF features from the soybean image dataset.
3. Apply the BoF algorithm to individually constructing the L*a*b*- and SURF-related visual dictionary by reducing the number of features through quantization of feature space using K-means clustering;
4. Form the hybrid semantic information by concatenating the L*a*b*- and SURF-related visual dictionaries.
5. Employ the LRR method to project the multiplicative-dimensional visual dictionary to low-rank space to eliminate redundant semantic information.
6. Perform the SVM algorithm to constructs the splitting hyperplanes in the high-dimensional kernel space to divide the jointly LRR data into three categories.

## Results and Discussion

### Color feature evaluation

As shown in Fig. 1, those appearance color properties of three kinds of soybean seed kernels differ from each other. The exposed yellow colors of the cotyledon-exposed soybeans (Fig. 1(b1,b2,b4)) are brighter than the good ones. The cotyledon surface colors of the naturally cracked soybean (Fig. 2(b3)) are a little bit more white and darker. The images of other unhealthy beans (Fig. 2(b5–10)) are mixed with other color features. Thereby, the color features can be adopted to discriminate the quality of soybean seeds. The collected raw soybean image data are stored in the RGB color format by using the customized imaging device. The RGB color model is device-dependent, which is initially designed to model the output of physical display or data acquisition devices. The image classification of soybean seeds is actually based on the human visual comprehensive perception of soybean seed color features. The L*a*b* color space mimics the nonlinear response of the eye. It can preserve the broad gamut of the color features of soybean seed image. All of the color which can be perceived by human eyes can be discovered in the L*a*b* color model^25^. The distribution of color features in the L*a*b* color space is more uniform than the RGB (see the light part in Fig. 2(c3)). The RGB color space contains too many transitional colors between blue and green, and lacks yellow and other colors between green and red (see Fig. 2(b1–3,c1–3)). As shown in Fig. 2(b1–3), the components of the RGB color space have small differences between their intensity values, so the visual perception of three components of RGB soybean image is extremely close. After color space conversion from RGB to L*a*b*, the components of L*a*b* color space have a significant difference between three distinct color channels, so Fig. 2(c1–3) show three extremely distinct types of images of visual perception effect. It enables the algorithm to easily quantify the visual differences between colors because it is more consistent with the Euclidean space structure. The converted distinguishable features are more suitable for the subsequent color dictionary generation procedure, which is based on k-means^32^. In order to more accurately express the color feature of soybean seed kernel images, we convert the RGB color descriptors to the CIEL*a*b*.

### SURF feature evaluation

**Figure 5:**
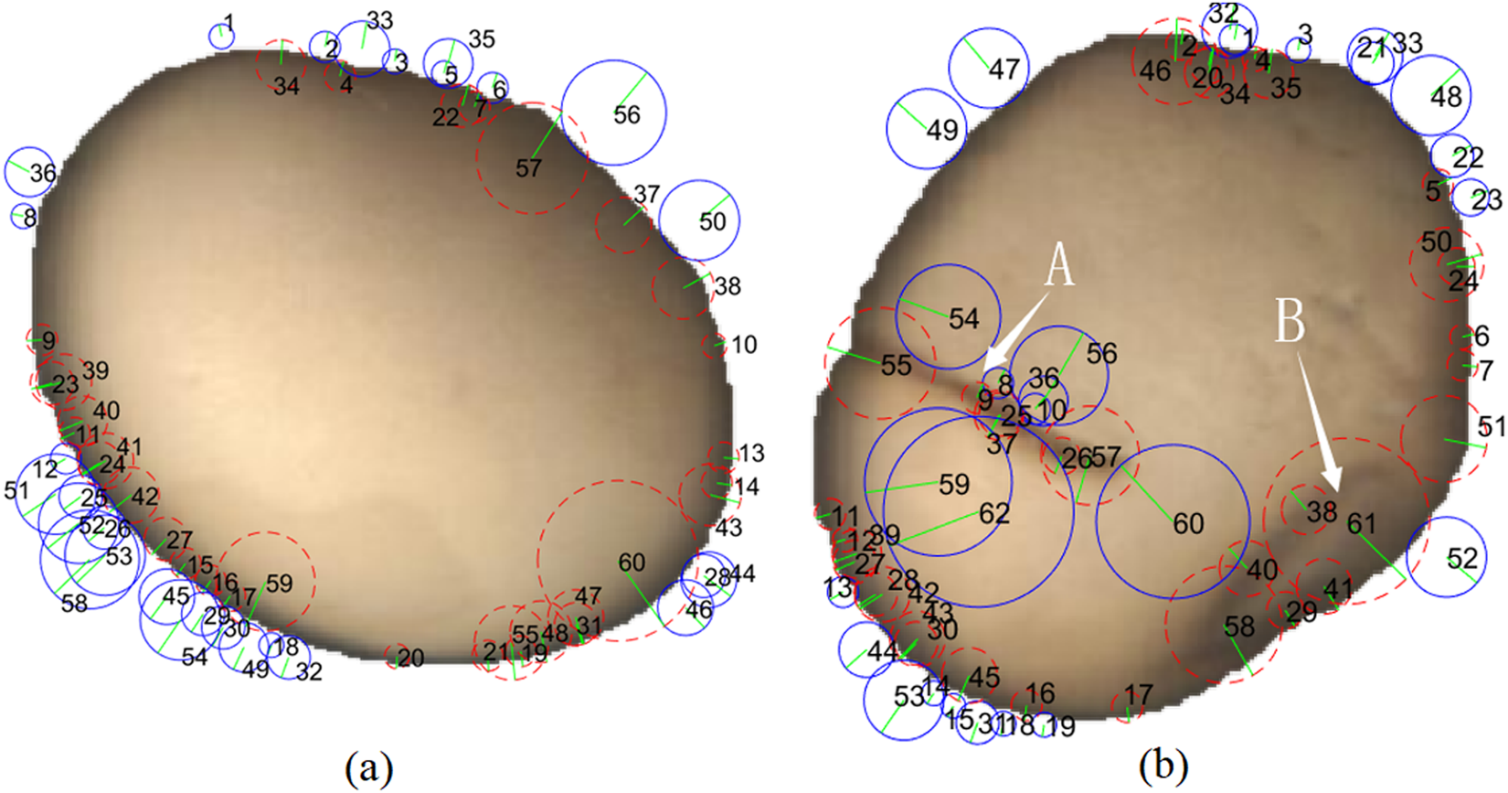
Comparison of the distribution of detected interest points of (**a**) good and (**b**) defective soybean seed images by using the SURF algorithm. The detected feature points are mainly distributed at the edge of the soybean image, as well as the cracked (at location A) and shriveled (at location B) region. The feature point areas are marked with the blue solid (Laplacian = −1) or red broken line ((Laplacian = +1)) circulars. The green radius indicates the dominant direction. Each feature point is numbered.

The SURF algorithm can estimate the placement angle by measuring the dominant orientation from the image. The soybean kernels are arbitrarily placed in the imaging panel, however, the extracted effective features are not affected by the placement angle, because the SURF feature is invariant to image rotation. As shown in Fig. 5(a), there are a total of 60 feature points detected by the SURF algorithm. The detected feature points are mainly distributed at the edge of the soybean image. The category of feature points corresponds to the sign of the Laplacian^27^. The Laplacian detected on the outside edge such as at the locations of 1, 46, 56 and 58 is −1, which are marked with blue solid circles; the Laplacian on the inside edge such as at the locations of 34, 57, 59 and 60 is +1, which are marked with red broken circles; Each feature point is numbered. The green radius indicates the dominant direction. The dominant direction is related to the feature area. The dominant direction around the inner and outer edge area is perpendicular to the tangential direction of the soybean outline. The surface color of good soybean kernel is relatively smooth and uniform, so the gradient change is relatively small, while the color around the edge area changes significantly, so the gradient change at the edge is larger relative to the inner smooth area. The SURF algorithm is mainly based on the gradient algorithm, so the characteristic points are detected on the edge of the soybean kernel.

As shown in Fig. 5(b), there are a total of 62 feature points detected by the SURF algorithm. Each feature point is numbered. The detected feature points mainly distribute in the edge, cracked (see the location A in Fig. 5(b)) and shriveled (see the location B in Fig. 5(b)) area of soybean kernel. The Laplacian detected on the outside of the edge such as at the locations of 47, 48, 52 and 53 is −1, which are marked with blue solid circles; the Laplacian on the inside edge such as at the locations of 11, 16, 35 and 51 is +1, which are marked with red broken circles; the detection center such as at the locations of 9, 26 and 57 has a Laplacian of 1 at the cracked area, which marked with red broken circles; the detection center such as at the locations of 38 and 40 has the Laplacian value of +1 in the shriveled area, which are marked with red broken circles. In the cracked and shriveled area, the dominant directions are approximately perpendicular to the cracked and shriveled direction, respectively. The darker image regions formed at the locations of the cracked and shriveled area relative to the yellow background region, so the gradient change at the edge is relatively large at these sites. The SURF method is mainly based on the gradient algorithm, so these feature locations are obviously perceived. The gradient-related information at the detected cracked and shriveled area can be used as the distinguishing feature for estimating the soybean quality.

### Jointly multi-modal feature evaluation

**Figure 6:**
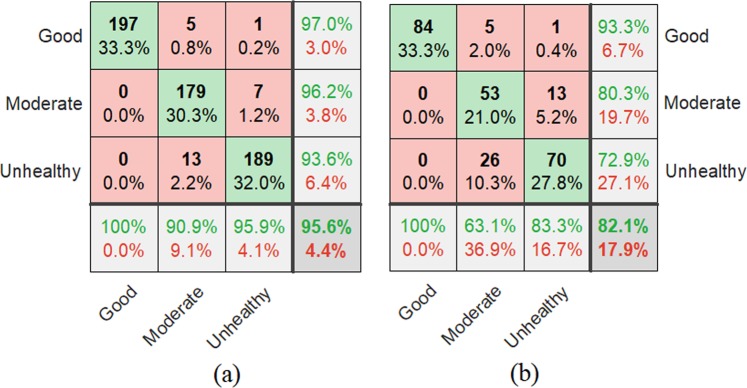
Summary of classification results of discriminating 3 kinds of good, moderate and unhealthy soybean seeds in terms of their appearance quality using the JMBoF + SVM model on the training (**a**) and test (**b**) dataset by using Confusion matrix graphics.

The confusion matrix diagram^33^ is utilized to summarize and visualize the results of the performance of the proposed JMBoF + SVM algorithm. As shown in Fig. 6, the rows of indicate confusion matrix the predicted results and the columns show the actual results. The correct classification results are shown on the green diagonal cells. For the training set, 197, 179 and 189 objects are correctly identified as the good, moderate and unhealthy soybeans, respectively. These correspond to 33.3%, 30.3% and 32.0% of all 591 training soybean instances, respectively. Similarly, for the test set, 84, 53 and 70 objects are correctly classified as good, moderate and unhealthy instances, respectively. These correspond to 33.3%, 21.0% and 27.8% of all 252 test soybean images, respectively. The red non-diagonal elements show where the model has made the wrong prediction. For the training set, 5 moderate and 1 unhealthy species are incorrectly classified as the good species, which correspond to 0.8% and 0.2% of all 591 good instances, respectively. 7 unhealthy species are mistakenly considered as the moderate species, which correspond to 1.2% of all 591 good instances. 13 moderate species are incorrectly classified as the unhealthy species, which correspond to 2.2% of all 591 good instances. Similarly, for the test set, 5 moderate and 1 unhealthy species are incorrectly classified as the good species, which correspond to 2.0% and 0.4% of all 252 soybean instances, respectively. 13 unhealthy species are incorrectly classified as the moderate samples, which correspond to 5.2% of all 252 soybean instances. 26 moderate species are incorrectly classified as the unhealthy species, which correspond to 10.3% of all 252 soybean instances. Out of 203 good predictions, 97.0% are correct and 3.0% are wrong. Out of 186 moderate predictions, 96.2% are correct and 3.8% are wrong. Out of 202 unhealthy predictions, 93.6% are correct and 6.4% are wrong. Out of 197 good cases, all are correctly predicted as the good species. Out of 197 moderate cases, 90.9% are correctly classified as the moderate species and 9.1% are classified as the good and unhealthy species. Out of 197 unhealthy cases, 95.9% are correctly classified as unhealthy and 4.1% are classified as the good and moderate species. Similarly, for the test set, out of 90 good predictions, 93.3% are correct and 6.7% are wrong. Out of 66 moderate predictions, 80.3% are correct and 19.7% are wrong. Out of 96 unhealthy predictions, 72.9% are correct and 27.1% are wrong. Out of 84 good cases, all are correctly predicted as the good species. Out of 84 moderate cases, 63.1% are correctly classified as the moderate and 36.9% are classified as the good and unhealthy. Out of 84 unhealthy cases, 83.3% are correctly classified as the unhealthy and 16.7% are classified as the good and moderate. Overall, 95.9% and 82.1% of the predictions are true and 4.4% and 17.9% are false on the soybean training and test set, respectively.

### Comparing the performance of algorithms

**Figure 7:**
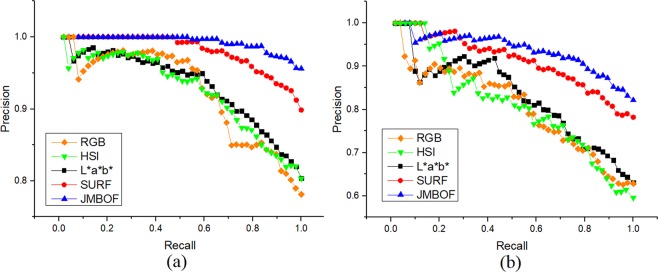
Precision-recall curves of discriminating three kinds of good, moderate and unhealthy soybean seeds in terms of their appearance quality using the methods of RGB + BoF + SVM, HSI + BoF + SVM, L*a*b* + BoF + SVM, SURF + BoF + SVM and JMBoF + SVM on the training (**a**) and test (**b**) dataset, respectively.

Precision-recall (PR) curve is used for visualization of evaluating the classifier performance. The precision indicates the true positive rate, while the recall indicates the probability of true positives in relation to all positive predictions^34^. Figure 7 shows the tradeoff between the precision and recall for different thresholds, namely the tendency for the recall to increase as the precision to decline. It is obvious that the JMBoF + SVM model basically holds higher percentage of precision rates than the RGB + BoF + SVM, HSI + BoF + SVM, L*a*b* + BoF + SVM and SURF + BoF + SVM models at the different thresholds of recall. The mean average precision (mAP) score which is the area under the precision-recall curve^35^ can be used as the integrated evaluation of algorithm performance. The JMBoF + SVM model yields the highest mAP scores of 0.973 and 0.918 on the training and test soybean image dataset, respectively, and outperforms the RGB + BoF + SVM of 0.904 and 0.787, HSI + BoF + SVM of 0.905 and 0.791, L*a*b* + BoF + SVM of 0.914 and 0.811 as well as the SURF + BoF + SVM of 0.962 and 0.893 (See Table 1).

**Table 1:** Accuracy and mean average precision (mAP) scores of grading 3 kinds of good, moderate and unhealthy soybean seeds in terms of their appearance quality using the RGB + BoF + SVM, HSI + BoF + SVM, L*a*b* + BoF + SVM, SURF + BoF + SVM and JMBoF + SVM models on the training and test dataset, respectively.

Method	Training set		Test set	
	Accuracy (%)	mAP	Accuracy (%)	mAP
RGB + BoF + SVM	78.1	0.904	62.8	0.787
HSI + BoF + SVM	80.3	0.905	59.5	0.791
L*a*b* + BoF + SVM	80.4	0.914	63.1	0.811
SURF + BoF + SVM	89.8	0.962	78.2	0.893
JMBoF + SVM	**95.6**	**0.973**	**82.1**	**0.918**

As shown in Table 1, the RGB + BoF + SVM model results in 78.1% and 62.8% accuracy, HSI + BoF + SVM model results in 80.3% and 59.6% accuracy, and L*a*b* + BoF + SVM model results in 80.4% and 63.1% accuracy on the training and test soybean image dataset, respectively. The L*a*b*-based single-modal algorithm outperforms the other two. It might be because several discriminated color features of soybean samples can be highlighted in the L*a*b* color space (see Fig. 2). The color-based single-modal method mainly distinguishes the soybean classes according to the overall characteristics of appearance. The damaged parts of defective soybean sometimes occupy a small proportion of the soybean surface. The corresponding extracted features also occupy a small ratio in the whole feature vector. It will result in the discriminated information ignored during the following classification process, so the low recognition rate is generated. Unlike the RGB, HSI and L*a*b*-based global color model, the SURF + BoF + SVM model does not apply the global color information from the soybean image. It attempts to detect the potential characteristic points and construct the gradient-based descriptor from the interest region. These feature points are mainly distributed at the edge and defective parts of the soybean kernel. Due to gaining the effective discriminated features, the SURF + BoF + SVM model improves the classification accuracy, which is 9.4% and 15.1% higher than the L*a*b* + BoF + SVM model on the training and test soybean image dataset, respectively. However, one potential drawback of this approach is that the relationships between the defective and intact patches and global image structure are ignored. This can be partially compensated by sampling the global L*a*b* features from the whole images. The JMBoF + SVM model takes advantage of local interest-region gradient-based features and global color feature information to further improve the classification accuracy. The L*a*b* + BoF + SVM model results in the highest 95.6% and 82.1% classification accuracy on the training and test soybean image dataset, which is 5.7% and 3.9% better than the SURF + BoF + SVM model, respectively.

## Conclusions

The paper firstly evaluates the appearance color properties for classifying the soybean seed kernels. The visual perception of three components of the RGB soybean image is extremely close. After color space conversion from RGB to L*a*b*, the components of L*a*b* color space show the significant visual difference between three distinct color channels. The extremely distinct types of images of visual perception effect enable to easily form distinguished features. The SURF feature is invariant to image rotation. The SURF algorithm can estimate the placement angle byout measuring the dominant orientation from the image. Though the soybean kernels are arbitrarily placed in the imaging panel, the extracted effective features are not affected by the placement angle. The dominant direction is related to the feature area. The dominant direction around the inner and outer edge area is perpendicular to the tangential direction of the soybean outline. In the cracked and shriveled area, the dominant directions are approximately perpendicular to the cracked and shriveled direction, respectively. The gradient change at the edge is larger relative to the inner smooth area, and the darker image regions formed at the locations of the cracked and shriveled area relative to the yellow background region, so the gradient change at the edge is relatively large at these sites. The SURF method is mainly based on the gradient algorithm, so these feature locations are obviously perceived.

Five different algorithms of RGB + BoF + SVM, HSI + BoF + SVM, L*a*b* + BoF + SVM, SURF + BoF + SVM and JMBoF + SVM are applied to classification of soybean quality. The multi-modal-based method of JMBoF + SVM outperforms than the other four single-modal-based algorithms, probably because the JMBoF + SVM model synthetically takes advantage of the global color feature and local interest-region gradient-based features (SURF) information. The JMBoF + SVM model results in the highest 95.6% and 82.1% accuracy on the training and test soybean image dataset, respectively. The proposed algorithm has the potential to be applied to the intelligent automated soybean grading machines for exactly, non-destructively and fast screening out the poor kernels. In the further, we intend to merge more effective discriminated feature elements from the soybean appearance to boost the accuracy of the classification algorithm.
